# *Bothrops jararaca* and* Bothrops erythromelas* Snake Venoms Promote Cell Cycle Arrest and Induce Apoptosis via the Mitochondrial Depolarization of Cervical Cancer Cells

**DOI:** 10.1155/2016/1574971

**Published:** 2016-12-06

**Authors:** Emanuelly Bernardes-Oliveira, Dayanne Lopes Gomes, Gustavo Martelli Palomino, Kleber Juvenal Silva Farias, Wilmar Dias da Silva, Hugo Alexandre Oliveira Rocha, Ana Katherine Gonçalves, Matheus de Freitas Fernandes-Pedrosa, Janaina Cristiana de Oliveira Crispim

**Affiliations:** ^1^Programa de Pós Graduação em Ciências Farmacêuticas, Universidade Federal do Rio Grande do Norte, Natal, RN, Brazil; ^2^Programa de Pós Graduação em Ciências da Saúde, Universidade Federal do Rio Grande do Norte, Natal, RN, Brazil; ^3^Laboratório de Imunoquímica, Instituto Butantan, São Paulo, SP, Brazil; ^4^Departamento de Tocoginecologia, Universidade Federal do Rio Grande do Norte, Natal, RN, Brazil; ^5^Maternidade Escola Januário Cicco (MEJC), Natal, RN, Brazil

## Abstract

*Bothrops jararaca* (BJ) and* Bothrops erythromelas* (BE) are viper snakes found in South-Southeast and Northeast regions of Brazil, respectively. Snake venoms are bioactive neurotoxic substances synthesized and stored by venom glands, with different physiological and pharmacological effects, recently suggesting a possible preference for targets in cancer cells; however, mechanisms of snakes have been little studied. Here, we investigated the mechanism responsible for snake crude venoms toxicity in cultured cervical cancer cells SiHa and HeLa. We show that BJ and BE snake crude venoms exert cytotoxic effects to these cells. The percentage of apoptotic cells and cell cycle analysis and cell proliferation were assessed by flow cytometry and MTT assay. Detection of mitochondrial membrane potential (Rhodamine-123), nuclei morphological change, and DNA fragmentation were examined by staining with DAPI. The results showed that both the BJ and BE venoms were capable of inhibiting tumor cell proliferation, promoting cytotoxicity and death by apoptosis of target SiHa and HeLa cells when treated with BJ and BE venoms. Furthermore, data revealed that both BJ venoms in SiHa cell promoted nuclear condensation, fragmentation, and formation of apoptotic bodies by DAPI assay, mitochondrial damage by Rhodamine-123, and cell cycle block in the G1-G0 phase. BJ and BE venoms present anticancer potential, suggesting that both* Bothrops* venoms could be used as prototypes for the development of new therapies.

## 1. Introduction

Cervical cancer is the third most common cancer in women worldwide [1, 2] and the fourth major cause of cancer death in women in developing countries, remaining a critical public health problem [3, 4]. In Brazil, it is estimated that there are 16,340 new cases of cervical cancer in 2016 [5]. High-risk human papilloma viruses (HPVs) such as HPVs 16, 18, 31, and 33 have been attributed to being the major risk factors for cervical cancer, out of which HPVs 16 and 18 account for almost 70% of the cancers [6, 7].

The method currently used in clinical medicine against different types of cancers, including cervical cancer, is surgical removal of the tumor followed by radiotherapy and chemotherapy [8]. Research on cancer is focused on discovery of new potential therapies, since the traditionally used drugs, such as Cisplatin (CDDP) and 5-Fluorouracil (5-FU), are often nonspecific and do not act directly on the tumor microenvironment. Therefore, new treatments for various types of cancers, including cervical cancer [9, 10], are considered one of the greatest challenges to medicine today because of the resistance to the effects due to repeated exposure [11]. Interventions with the use of chemotherapy are far from satisfactory, because of side effects, destruction of healthy cells, and above all acquired resistance by tumors [12–14].

Anticancer therapy is one of the main areas for the use of proteins and peptides originating from animals. Some of these proteins or peptides, when isolated, may bind specifically to cancer cell membranes, affecting the migration and proliferation of these cells. Venoms and toxins from snakes may hold the promise for treating many types of malignancies, especially with the demonstration of complete remission of cancer cells after treatment with molecules derived from animal venom. However, studies focusing on the mechanisms by which these venoms act are still very recent, and much has yet to be found out about these molecules [15].

Some approaches with snake venoms have been of great importance in the presentation of anti-inflammatory activity [16], antibacterial activity [17], and antiparasitic activity against* Leishmania* [18], making it a natural source of interest to cancer therapy [19, 20]. Previous trials have reported that snake venoms are able to act on the tumor in some models, such as melanoma (B16F10 cells) [21], breast (MCF-7 cells) [22], colon (HCT116 and HT-29 cells) [23], lung cancer (NCL-H460 cells) [24], and neuroblastoma (SK-N-MC and SK-N-SH cells) [25]. However, despite these data, there are few studies relating to* Bothrops* in cervical cancer cell lines. In this approach, the cervical cancer cell lines SiHa (HPV 16) and HeLa (HPV 18) were subjected to treatment with the venoms of snakes* Bothrops jararaca* and* Bothrops erythromelas*.

By virtue of the few studies exploring the venoms snake and cervical cancer, the aim of this study was to evaluate the antitumor action of species* Bothrops* snake venoms in tumor cell lines* in vitro* SiHa and HeLa in a concentration-dependent manner.

## 2. Materials and Methods

### 2.1. Reagents

The following reagents were purchased as indicated: 4,6-diamidino-2-phenylindole (DAPI), 3-(4,5-dimethylthiazol-2-yl)-2-5-diphenyltetrazolium bromide (MTT), 2-(6-amino-3-imino-3H-xanthen-9-yl) benzoic acid methyl ester (Rhodamine-123), sodium pyruvate and essential amino acids, trypsin, and dimethyl sulfoxide (DMSO) were purchased from Sigma Chemical Company (St. Louis, MO, USA). Dulbecco's modified Eagle's medium (DMEM) and fetal bovine serum (FSB) were obtained from Cultilab (Campinas, SP, Brazil). Annexin V-FITC and propidium iodide (PI) were used for flow cytometry (Invitrogen). Cisplatin (citoplax, 50 mg from Bergamo Taboão da Serra, SP, Brazil).

### 2.2. Venom and Treatments

The crude venoms of* B. jararaca* (BJ) and* B. erythromelas* (BE) were kindly supplied by the Butantan Institute, São Paulo, Brazil. All solutions were filtered using a 0.22 *μ*m of minipore membrane and then were aliquoted and stored at 20°C.

### 2.3. Cell Lines

SiHa human squamous cell carcinoma HPV-16-positive and HeLa cervical adenocarcinoma cells HPV-18-positive were donated by Dr. Ana Paula Lepique (Department of Immunology, Universidade de São Paulo, Brazil) and nontumor cells of mouse embryo 3T3 were from the Department of Biochemistry (Universidade Federal do Rio Grande do Norte, Natal, Brazil).

### 2.4. Cell Culture and MTT Colorimetric Assay

Cell lines SiHa, HeLa, and 3T3 were cultured with DMEM, supplemented with 10% FBS and 1% antibiotic, sodium pyruvate, and essential amino acids, in an incubator containing 5% CO_2_, and maintained at 37°C and the culture media were changed as needed. The cytotoxicity was investigated by MTT assay. Cells were seeded in a 96-well plate at an initial density of 5 × 10^3^ cells per well in DMEM complete medium and 10% FBS, incubated with 5% CO_2_ at 37°C for 24 h and 48 h. Then, the BJ, BE, and CDDP (drug control) were added whose concentrations varied from 12.5 *μ*g/mL, 25 *μ*g/mL, and 50 *μ*g/mL, 33 *μ*g/mL, respectively. Concentrations were used in triplicate. After 24 h and 48 h incubation, MTT dyes 50 *μ*L (1 mg/mL) were added to the wells and incubated for 4 h. The MTT-formazan product dissolved in 100 *μ*L of DMSO PA was estimated by measuring the absorbance at 540 nm in a Multiskan Ascent Microplate Reader (Thermo LabSystems, Franklin, MA, USA). The absolute value of MTT reduction was calculated as follows:

(1) $$\begin{array}{c} \text{MTT reduction}=\frac{\text{Abs.}\;\;540\;nm\;\;of\;\;sample}{\text{Abs.}\;\;540\;nm\;\;of\;\;control}. \end{array}$$

### 2.5. Annexin V-FITC/PI Double Staining and Analysis by Flow Cytometry

To evaluate the effects of BE and BJ on cell death, the SiHa and HeLa cells were seeded (2 × 10^5^ cells) per well in 6-well plates, after treatment with 50 *μ*g/mL of* Bothrops* venoms and 33 *μ*g/mL CDDP for 48 h and were incubated with 5 *μ*L annexin V-FITC and 1 *μ*L propidium iodide (PI) for 15 min at room temperature following kit directions (Invitrogen, Catalog number V13242). The cells were analyzed by flow cytometry (flow cytometer FASCANTO II, BD Biosciences), measuring fluorescence emission at 530–575 nm for annexin V and 630/22 nm for PI. 10.000 events were acquired. The FlowJo software version X10.0.7 (Tree Star, Inc., Ashland, OR, USA) was used for data analysis as described in Gomes et al., 2015 [26].

### 2.6. Analysis of Mitochondrial Membrane Potential (MMP) by Rhodamine-123 (Rh-123) Staining

MMP in SiHa cells was measured by fluorochrome Rh-123. SiHa cells were cultured in the six-well plates (2 × 10^6^ cells/mL); the cells were then treated for a further 48 h with venoms BJ and BE, as described previously. Cells were harvested and washed twice with PBS, then incubated with 0.5 *μ*L Rh-123 (5 mg/mL diluted in ethanol) staining solution at 37°C in the dark for 15 min, then washed twice with PBS, and centrifuged at 500 ×g for 30 min and were analyzed by a FACSCalibur flow cytometer.

### 2.7. DAPI Staining

For morphological analysis, SiHa cells were seeded on cover slips at a density of 3 × 10^4^ cell wells in 24-well plates. After 48 h of incubation with BJ and BE venoms at 50 *μ*g/mL, treated cells were washed twice with cold PBS and were fixed with 4% paraformaldehyde for 30 min. Then, the cells were permeabilized with 0.1% Triton X-100 at room temperature for another 30 min. Samples were subsequently incubated in DAPI (1 *μ*g/mL) solution at room temperature for 30 min, washed with PBS, and examined under a fluorescence microscope.

### 2.8. Cell Cycle Analysis

SiHa cells were plated in a six-well plate (2 × 10^5^ cells/mL) and were then stimulated to enter G0 in a medium without serum for 24 h. Next, cells were to exit G0 by adding DMEM supplemented with 10% FBS, in the presence of BJ and BE (50 *μ*g/mL) or CDDP (33 *μ*g/mL). After 48 h, the cells were harvested, washed twice with cold PBS, and centrifuged; cell pellets were incubated in 2% paraformaldehyde for 30 min, washed with cold PBS, and permeabilized with 0.01% saponin for 15 min. After this procedure, cells were incubated with 10 *μ*L of RNase (4 mg/mL) at 37°C for 45 min. Two microliters of PI and 200 *μ*L of PBS were added protected from light. The DNA content was analyzed using a FACSCalibur flow cytometer. A total of 30.000 events were acquired. For data analysis, FlowJo software version X10.0.7 was used (Tree Star, Inc., Ashland, OR, USA). Data presented are representative of those obtained in at least three independent experiments done in duplicate.

### 2.9. Statistical Analysis

Each experiment was performed at least 3 times. We used analysis of variance (ANOVA) and Tukey's *t*-test. Differences with *p* < 0.001 between the values are considered statistically significant. Statistical analysis and the Pearson correlation coefficient (*ρ*) were performed using GraphPadInStat® software version 4.0 (GraphPad software, San Diego, CA, USA).

## 3. Results

### 3.1. Morphological Changes of SiHa and HeLa Cells after Venom of Snakes

Morphological changes were analyzed by optical microscopy after venom of snakes treatment, compared with the cell without any treatment.

**Figure 1:**
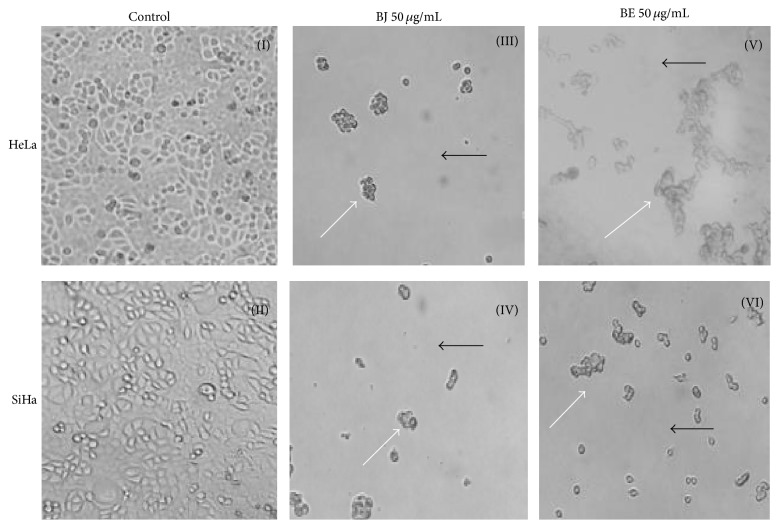
Morphology suggests cytotoxicity of* Bothrops* snake venoms in HeLa and SiHa cells (40x), after 48 h of* B. jararaca* (BJ) and* B. erythromelas* (BE) treatment. (I) and (II) (untreated cells), (III) and (IV) (BJ 50 *μ*g/mL), and (V) and (VI) (BE 50 *μ*g/mL).

As shown in Figure 1, HeLa and SiHa cells (I and II) without any treatment show well bonded growth on the plate, with fusiform with high refractive index and straightforward cytoplasm. However, in cells HeLa (II and V) and SiHa (IV and VI), after being treated with BJ and BE (50 *μ*g/mL) and cultured for an additional 48 h, the cells gradually become rounder (white arrow). Morphological observation indicated that the cells were gradually reduced in size; there is a detachment of the cell monolayer (black arrow) suggesting a significant reduction in the amount of cells when compared to untreated cells.

### 3.2. The Effect of the* Bothrops* Venom Cytotoxicity on Cervical Cancer Cells

**Figure 2:**
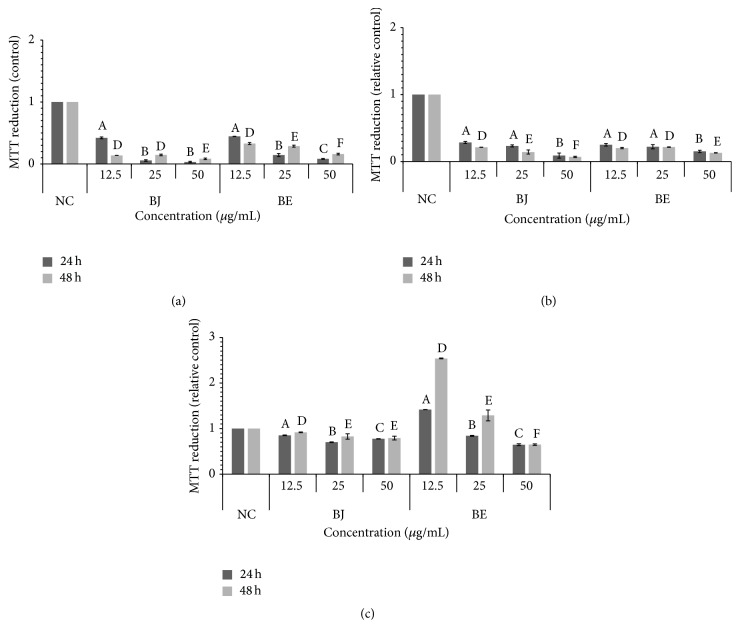
Cytotoxicity of* Bothrops* snake venoms. SiHa (a), HeLa (b), and 3T3 (c) cells. Untreated cells (NC),* B. jararaca* (BJ), and* B. erythromelas* (BE). Cisplatin was used as a control drug. Different letters indicate a significant difference between different concentrations of the* Bothrops* venoms, A, B, and C (24 h) and D, E, and F (48 h) *p* < 0.001.

Through MTT assay, the cytotoxicity was analyzed focusing on* B. jararaca* and* B. erythromelas* venom in tumor cell lines SiHa and HeLa cervical cancer cells and normal 3T3 line. Cells were treated with various concentrations of BJ and BE (12.5, 25, and 50 *μ*g/mL) for 24 and 48 h (Figure 2). It was observed that the absolute values for SiHa were 0.42 (BJ) and 0.44 (BE) at concentrations of 12.5 *μ*g/mL in both treatments for 24 h. When the cell was subjected to the same treatment during 48 h, there was a smaller reduction in the absolute values MTT 0.14 (BJ) and 0.33 (BE), suggesting the cytotoxicity of BJ and BE venoms presented similar concentrations (Figure 2(a)). For the same treatment in the HeLa cell line, at 24 h, the absolute values of 0.28 (BJ) and 0.25 (BE) were observed; however, at 48 h, these became 0.25 (BJ) and 0.20 (BE), revealing that these venoms can promote the reduction of the MTT cytotoxicity at a lower concentration (Figure 2(b)).

Unlike cervical cancer cells,* Bothrops* venoms were not cytotoxic to normal 3T3 fibroblast cell with absolute values of 0.70 (BJ) and 1.42 (BE), with respective 25 *μ*g/mL and 12.5 *μ*g/mL concentrations for 24 h. At 48 h, 0.92 (BJ) and 2.54 (BE) were obtained both at lower concentrations of 12.5 *μ*g/mL (Figure 2(c)). Note that these venoms have selective action on SiHa and HeLa cell lines.

### 3.3. Apoptosis Assay

To determine whether the cytotoxicity effects of* Bothrops* snake venoms are associated with cell death, we use cell death markers annexin V-FITC/PI in order to differentiate the cells undergoing apoptosis and necrosis after the treatment. The annexin V-labeled cells indicate apoptosis initial death, while PI is indicative of necrosis, but the cells positive for annexin V-FITC/PI are indicative of late apoptosis.

**Figure 3:**
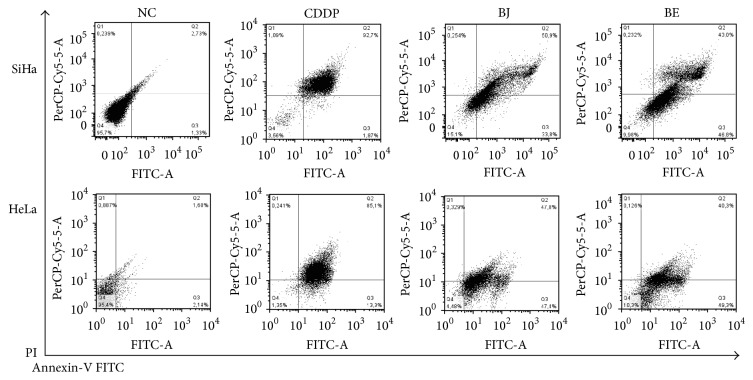
*Bothrops* snake venoms induce apoptosis SiHa in HeLa cells (48 h). Cervical cancer cells were treated with BJ and BE 50 (*μ*g/mL), with untreated cells compared and analyzed by annexin V-FITC/PI staining. Frames were divided into four quadrants: cells stained with annexin V and PI were classified as necrotic (Q1; annexin V−/PI+), late apoptotic (Q2; annexin V+/PI+), early apoptotic (Q3; annexin V+/PI−), or intact (Q4; annexin V−/PI−). Untreated cells (NC), Cisplatin (CDDP),* B. jararaca* (BJ), and* B. erythromelas* (BE).

This study showed that the death of SiHa cells presented 84.95% and 90.02%, respectively, when treated with BJ and BE. Similar results were obtained in HeLa cells, with a total kill of 95.52% (BJ) and 89.72% (BE). The same cells when untreated obtained a low death rate of 4.29% (SiHa) and 4.6% (HeLa). The analysis of flow cytometry was performed with the drug control CDDP (Figure 3).

### 3.4. Morphological Analysis with DAPI

**Figure 4:**
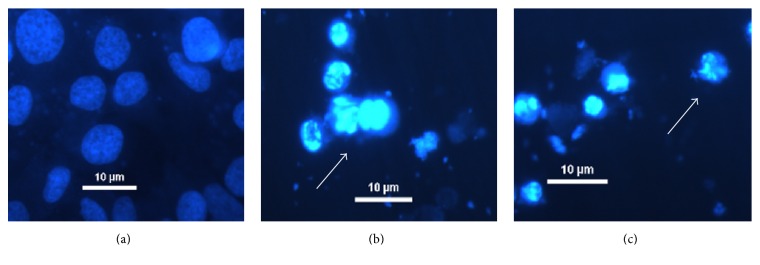
SiHa nuclei morphology after treatment with* Bothrops* venoms. Untreated cells (a),* Bothrops jararaca* (b), and* Bothrops erythromelas* (c). White arrows indicate the typical apoptotic cell (400x magnification).

In order to elucidate the relationship of death by apoptosis, we need to evaluate changes in the morphology of chromatin in cells using DAPI staining. In the untreated group, the SiHa cells were rounded and homogeneously stained (Figure 4(a)). After 48 h of treatment with the crude venoms (50 *μ*g/mL), the cells showed morphological changes such as chromatin condensation, the appearance of apoptotic bodies, and nuclear vesicles (Figures 4(b) and 4(c)).

### 3.5. Analysis of Mitochondrial Membrane Potential (MMP) by Fluorochrome Rhodamine-123 (Rh-123)

**Figure 5:**
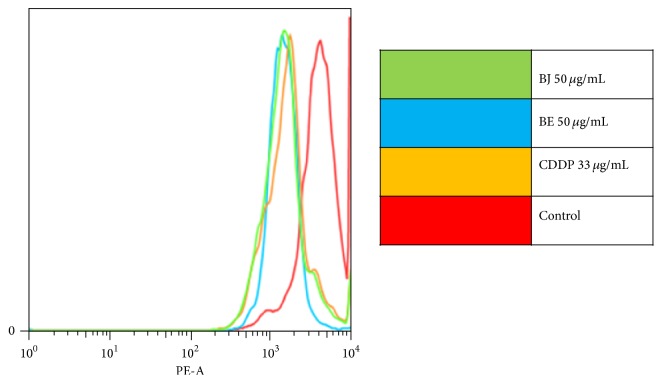
Reduced mitochondrial membrane SiHa cells after treatment with* Bothrops* venoms. Untreated cells (control), Cisplatin (CDDP),* B. jararaca* (BJ), and* B. erythromelas* (BE). Colors: green (BJ 50 *μ*g/mL), blue (BE 50 *μ*g/mL), yellow (CDDP 33 *μ*g/mL), and red (untreated cells).

For the potential analysis of MMP in SiHa posttreatment of BJ and BE cells (50 *μ*g/mL) and CDDP (33 *μ*g/mL), Rh-123 dye was used which gave a distribution histogram of the treatment groups for the purposes of comparison with the control group (untreated cells). For the mitochondrial membrane, potential analysis was obtained from distribution curves. Through the histogram (left shift), an inhibition was similarly observed in the function of mitochondria, with no differences between treatments. The results suggest that venoms may reduce the mitochondrial membrane potential of SiHa cells by depolarization inducing death by apoptosis via the mitochondrial pathway (Figure 5).

### 3.6. Cell Cycle Arrest

**Figure 6:**
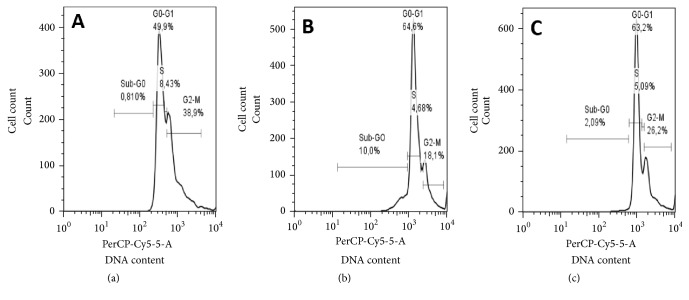
Cell cycle analysis of SiHa cells at 48 h. After treatment with BJ and BE (25 *μ*g/mL), cells were collected and stained with PI, and then flow cytometry analysis was performed. Representation of the dot plots obtained from flow cytometry. Untreated cells (a),* B. jararaca* (b), and* B. erythromelas* (c).

The antiproliferative activity of the crude venoms also demonstrated alteration of cell cycle progression in SiHa cells at a concentration of 25 *μ*g/mL. The BJ venom had its passage blocked in G0/G1 phase of the cell cycle 64.6% (Figure 6(b)). This effect was also observed with BE which blocked cell growth of the cell during the same phase (63.2%) (Figure 6(c)). The data indicate that the cells in G1 phase during incubation entering the subsequent stage are interrupted at this point. If the cells entered apoptosis from the G1 phase, there was no accumulation of cells in G2/M and S phase. The same parameters were not observed in untreated cells (Figure 6(a)).

## 4. Discussion

In the context of cancers, some approaches have been conducted with venoms and toxins from snakes. Recently, Tavares et al. (2016) observed the induction of cytotoxicity by L-amino acid oxidase activity from the venom of* Calloselasma rhodostoma* species in bone marrow erythroleukemia cell lines (HEL92.1.7 cells) and megakaryoblastic leukemia (Set-2 cells) [27]. Other studies with adenocarcinoma cell lines of human colon LS174 (p53wt), HCT116 (p53wt), and HT29 (p53mut) found that Lebein (disintegrin heterodimeric) isolated from the venom of* Macrovipera lebetina* snake significantly inhibited cell viability [28]. Like the venoms of snakes of the genus* Bothrops*, the species* B. mattogrossensis* had three isolates of phospholipase A2 (PLA2) with cytotoxic activity in Jurkat lines (leukemia T) and SK-BR-3 (breast adenocarcinoma) [29]. But it is noteworthy that so far there are rare works in the literature exploring cervical carcinoma cell lines SiHa and HeLa, challenged with the venoms of* B. jararaca* and* B. erythromelas* snakes.

After treatment with* Bothrops* venoms, the first step was to analyze the cell morphology by optical microscopy; this revealed that both crude venoms have antitumor activity in tumor cell lines and found that BJ and BE promoted reduction of the cell size making them rounded. These findings corroborate with Lee et al. [2], when they tested the toxin* Vipera lebetina turanica* snake in carcinoma cells.

To confirm this finding, an MTT reduction assay was performed in which only metabolically active cells had the ability to reduce it. The result of this assay showed the reduction of MTT to absolute values of 0.03 and 0.07 of BJ and BE, respectively, thus confirming the cytotoxicity of these venoms in SiHa and HeLa cell lines. According to Aranda-Souza et al. [30], a reduction was also observed of melanoma cells (B16-F10) when treated with a lectin extracted BIL in* B. leucurus* snake venom. When Nunes et al. [31] used BIL venom of the same species* B. leucurus* in K562 cells (erythromyeloblastoid human leukemia cell line), it showed the cytotoxic activity, while HaCaT cells (normal cell) were not affected. In order to confirm this same action of BJ and BE in normal cells, we used 3T3 cells, which verified that the tested venoms were not cytotoxic.

This fact is very important since one of the most important problems occurring in chemotherapeutic interventions today is the nonspecificity of the compound for tumor cells and/or destruction of healthy cells; for example, CDDP used in this study decreased the viability of normal cells (3T3) by more than 50% (data not shown). Therefore, we suggest that BJ and BE could become a promising treatment for cancer cells of the cervix, with a similar toxicity to drug CDDP control. Given this result, it is interesting to test BJ and BE with other normal cell lines such as human fibroblasts.

To verify what type of cell death was induced by BJ and BE, SiHa and HeLa were incubated with annexin V-FITC/PI after treatment with venoms, in which, cells labeled with annexin indicate early apoptosis, cells labeled with PI are indicative of necrosis, and cells positive for annexin and PI are indicative of late apoptosis. Data suggests that all cells that had their viability affected by venoms had death induced by apoptosis, with 50% at the initial apoptosis and 50% in the final stages of apoptosis, which makes this the most important finding because the cancer cells showed a reduced sensitivity towards apoptosis and tumors are dependent on the mechanisms of this resistance to persist and to continue development.

What is more, Prinholato da Silva et al. [32] demonstrated in different tumor cells HL-60 (promyelocytic leukemia), HepG2 (human hepatocellular carcinoma), PC-12 (murine pheochromocytoma), and B16F10 (murine melanoma) which BthTX-I toxin isolated from snake venom of* B. jararacussu* induced these cell deaths via apoptosis and/or necrosis in concentrations tested (25, 50, or 100 *μ*g/mL). While other studies have shown that snake venom toxins inhibited tumor growth accompanied with inactivation of nuclear factor kappa B (NF-*κ*B), thereby preventing human cervical cancer cell growth (CaSki and C33A) by the induction of apoptotic cell death [2].

Nolte et al. [33] showed that the lectin BJcuL extracted from the venom of* B. jararacussu* presented cytotoxic activity in gastric cancer cells (MKN45 and AGS), mainly by changing cell adhesion and providing the induction of apoptosis by death.

Research by Zhang and Wei [34] noted apoptotic changes such as phosphatidyl serine externalization signaled by positive labeling for annexin and DNA fragmentation of HeLa cell, a protein isolated from ACTX-8-treated* Agkistrodon acutus* snake venom. Therefore, the discovery of selective drugs that affect the balance of tumor cellular functions towards apoptosis is of enormous therapeutic interest.

The data can suggest that this cell death by apoptosis can occur via mitochondria, the organelle that is important in the regulation of this type of death. To better understand the action of BJ and BE, we analyzed the MMP by Rh-123 in SiHa cells that have the HPV type most prevalent among cervical cancers in women worldwide [35]. Through this dye, the main results confirmed that both* Bothrops* venoms decreased mitochondrial membrane potential when compared to untreated cells. Our results were similar to the findings of Rajeshkumar et al. [36] who found that the marine animal venom* Dasyatis sephen* depolarized the mitochondrion of HeLa cell.

Some studies have shown the damage to the DNA of the tumor cells by the action of* Bothrops* snake venoms. Research by Son et al. [37] observed in PC-3 cells shrinkage of these cells and formation of apoptotic bodies DU145 and LNCaP (prostate cancer). On the other hand, Gabriel et al. [38] studied leucurolysin B (leukocyte-B), which is a P-metalloproteinase class III isolate of* B. leucurus* in tumor cell lines T98 (p53 mutant malignant glioblastoma), U87 and RT2 (wild-type p53 malignant glioblastoma), MCF7 (breast carcinoma), EAC (Ehrlich ascites carcinoma), and UACC (melanoma) and viewed the chromatin condensation and nuclear fragmentation by DAPI staining, with results similar to ours.

Consequently, there was damage to the DNA of the cell where the cell cycle was interrupted. The cell cycle is a series of processes that leads to cell division to enter. When these cells mutate, it can die by apoptosis or divide without control as with tumor cells. To better understand the damage to the DNA of SiHa cell, the cell cycle was analyzed and it was found that BJ and BE were able to induce a change in the cell cycle promoting prevention at G0/G1 phase and not allowing the tumor cell to enter mitosis. The main findings of this article are similar to Prinholato da Silva et al.'s [32], who examined the cell cycle arrest in PC-12 (murine pheochromocytoma) and B16F10 (murine melanoma) tumors when treated with BthTX-I* B. jararacussu*.

However, specific approaches are needed in the future to support this assumption and to identify its mechanism of action in modulating death in human cervical cancer cell. In addition, the significance of the observations made in the present study needs to be established in a broader context by conducting further studies with a variety of techniques regarding human cervical cancer.

## 5. Conclusions

Both snake venoms tested in this approach exhibited antitumor action according to the concentration-dependent dose. Given this result, advanced research is required to determine what exactly favors the venom to promote antitumor action. Therefore, it is suggested that studying the mechanisms involved in cell death becomes a priority. Our results indicated that snake venoms of* B. jararaca* and* B. erythromelas* present a significant potential application in cervical cancer therapy.
